# The role of menopause and reproductive senescence in a long-lived social mammal

**DOI:** 10.1186/1742-9994-6-4

**Published:** 2009-02-03

**Authors:** Eric J Ward, Kim Parsons, Elizabeth E Holmes, Ken C Balcomb, John KB Ford

**Affiliations:** 1 Northwest Fisheries Science Center, 2725 Montlake Blvd E, Seattle, WA 98112, USA; 2 Alaska Fisheries Science Center, 7600 Sand Point Way NE, Seattle, WA 98115, USA; 3 Center for Whale Research, PO Box 1577, Friday Harbor, WA 98250, USA; 4 Pacific Biological Station, Fisheries and Oceans Canada, Nanaimo, BC V9T 6N7, Canada

## Abstract

**Background:**

Menopause is a seemingly maladaptive life-history trait that is found in many long-lived mammals. There are two competing evolutionary hypotheses for this phenomenon; in the adaptive view of menopause, the cessation of reproduction may increase the fitness of older females; in the non-adaptive view, menopause may be explained by physiological deterioration with age. The decline and eventual cessation of reproduction has been documented in a number of mammalian species, however the evolutionary cause of this trait is unknown.

**Results:**

We examined a unique 30-year time series of killer whales, tracking the reproductive performance of individuals through time. Killer whales are extremely long-lived, and may have the longest documented post-reproductive lifespan of any mammal, including humans. We found no strong support for either of the adaptive hypotheses of menopause; there was little support for the presence of post-reproductive females benefitting their daughter's reproductive performance (interbirth interval and reproductive lifespan of daughters), or the number of mature recruits to the population. Oldest mothers (> 35) did appear to have a small positive impact on calf survival, suggesting that females may gain experience with age. There was mixed support for the grandmother hypothesis – grandoffspring survival probabilities were not influenced by living grandmothers, but grandmothers may positively influence survival of juveniles at a critical life stage.

**Conclusion:**

Although existing data do not allow us to examine evolutionary tradeoffs between survival and reproduction for this species, we were able to examine the effect of maternal age on offspring survival. Our results are consistent with similar studies of other mammals – oldest mothers appear to be better mothers, producing calves with higher survival rates. Studies of juvenile survival in humans have reported positive benefits of grandmothers on newly weaned infants; our results indicate that 3-year old killer whales may experience a positive benefit from helpful grandmothers. While our research provides little support for menopause evolving to provide fitness benefits to mothers or grandmothers, our work supports previous research showing that menopause and long post-reproductive lifespans are not a human phenomenon.

## Background

Individuals in many mammalian species devote a fraction of their lives to reproduction. Females in these species experience a gradual midlife decline in reproductive performance with age, and eventually menopause, the abrupt termination of reproduction [1]. In contrast, males are typically able to reproduce until old age, with only a slight decrease in reproductive ability. Although anthropologists have suggested that menopause is unique to primates [2] or even just humans [3,4], the phenomenon appears to be widespread in mammals – in a meta-analysis of 42 species, Cohen (2004) found support for post-reproductive lifespans (PRLS) in 83% of taxa. More recent studies have shown that there are a range of species with relatively little or no parental investment that also exhibit reproductive senescence and cessation, including species generally considered to be 'r-selected'. These species include guppies [5], parakeets [6], mice [7], and beetles [8].

Although reproductive senescence or menopause may be relatively common, only a handful of species have extremely long post-reproductive lifespans. Why do females of some species live so long after reproduction ceases? On the surface, this would appear to be a maladaptive life-history trait. Several hypotheses, adaptive and non-adaptive, have been proposed to explain the evolutionary significance of menopause.

### The attentive mother hypothesis

Under the adaptive view of menopause, the cessation of reproduction allows for an increase in individual or inclusive fitness [9]. This so-called "attentive mother" hypothesis predicts that as the level of required infant care increases, early reproductive termination allows females to provide a marginal benefit to the survival of existing offspring [9]. Older females that stop reproducing simultaneously avoid risky pregnancies. Of all primate species, evidence supporting the attentive mother hypothesis is limited to humans [10]. A variant of this theory, also only applicable to primates, is the "altriciality-lifespan" model. This hypothesis correlates brain size with parental care, suggesting that an increase in the age at the onset of menopause may be coupled with selection for long lifespans [3].

### The helpful grandmother hypothesis

A second adaptive theory of menopause is the grandmother hypothesis. By extending their lifespans after reproduction ceases, post-reproductive females who help daughters or other kin raise offspring increase their own inclusive fitness [10,11]. Specifically, females with living mothers may experience increased fecundity rates, decreased interbirth intervals (IBI), and increased reproductive lifespans [12,13]. Evidence supporting the grandmother hypothesis is inconclusive, and limited to humans. In a study of 18^th ^century humans, Lahdenpera et al. (2004) showed a positive effect of grandmothers on both grandchild survival to maturity and the reproductive lifespan of daughters. Additional studies of the grandmother hypothesis have failed to find support in other social species, including lions and baboons [12], macaques [14], and pre-industrial humans [15,16]. The lack of support for the grandmother hypothesis may be due to a lack of high quality longitudinal data, contradictory effects of maternal and paternal grandmothers [17], or complicated kin relationships [18].

### The mutation accumulation hypothesis

An alternative hypothesis to the adaptive views of menopause is the "mutation accumulation" model [9,19]. Under this hypothesis, senescence is a result of physiological deterioration associated with aging, and weak selection on aged individuals due to few animals living to extremely old ages. Menopause becomes a byproduct of this process, and is more evident when selection acts to increase longetivity, increasing the difference between the lifespans of the reproductive and somatic systems [20]. Support for the mutation accumulation hypothesis is generally indirect, consisting of rejecting competing hypotheses [12,21].

### Reproductive senescence and menopause in killer whales

**Figure 1:**
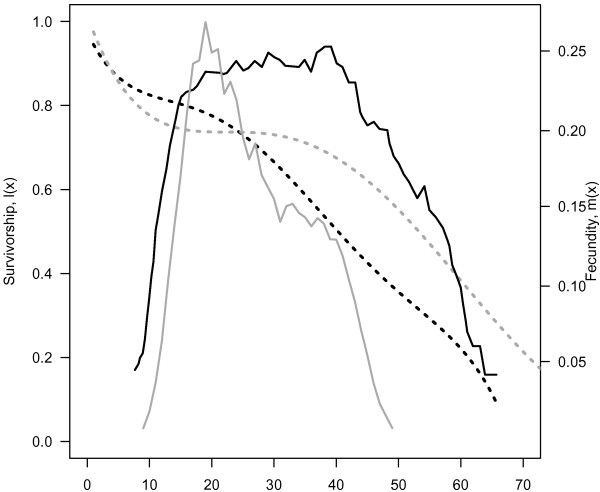
**Age specific survivorship (solid line) and fecundity (dashed line) for elephants (black**, [28]**) and killer whales (grey**, [22,26]**).** Survivorship calculations are sex-specific, shown for females only.

Killer whales represent a model organism for examining potential benefits of long lived mothers and grandmothers. Like humans, killer whales are extremely long lived, experience differential sex-specific mortality [22], and females have long post-reproductive lifespans. Few males reach 50 years of age, but female life expectancy is considerably longer (> 50 years, Figure 1). Although there is some uncertainty in the estimated ages of the oldest individuals, one female known as J2 is thought to be more than 90 years old [23,24]. This longevity is rare in mammals, and even greater than the maximum age observed in hunter-gatherer human populations [25]. Relative to elephants, which mature at a similar rate, killer whales are thought to have lower natural mortality, an increased rate of reproductive senescence, and spend a smaller fraction of their lifespan reproducing (Fig. 1; [26-28]). Females may produce their first calf as early as age 10, and continue to produce offspring until their early 40s [22]. Over this time, the mean interval between births averages 3.4 years (*σ*^2 ^= 5.6). Like other species with long post-reproductive lifespans, killer whale calves require high maternal investment. Before weaning (~1 year), calves are highly dependent, and do not survive in the absence of their mothers. Combined, these factors translate into a smaller growth rate and potentially the longest post-reproductive lifespan of any mammal [29,30].

One of the reasons that killer whales have been thought to support the grandmother hypothesis is that their social structure is matrilineal. Collections of matrilines – a mother and her offspring – associate with other matrilines, forming larger social aggregations of related individuals ('groups' or 'pods'). Among piscivorous killer whales (also known as 'residents'), males and females are both philopatric and remain with their natal matriline throughout their life (fathers contribute no parental care). From a kin-selection perspective, individuals in the same matriline engage in many of the same food sharing behaviors that have been linked to the grandmother hypothesis in human populations [10,31]. While it is not possible to quantify the benefit of food sharing for this species, additional mechanisms that may enable grandmothers to increase inclusive fitness may be providing additional care for newborns, or benefitting the social unit by retaining cultural information such as knowledge of historically good hunting areas [32,33].

The length of killer whale post-reproductive lifespans has led some authors to suggest that the grandmother hypothesis is responsible for the extreme longevity of females in this species [33], however support for the grandmother hypothesis has not been evaluated with data. The aim of this study was to quantify the data support for both adaptive views of menopause using long-term data tracking every individual killer whale. We used these data to estimate the effect of post-reproductive mothers and grandmothers on demographic rates, using data collected from two distinct populations of killer whales. First, we examined whether maternal covariates had positive effects on the survival of offspring. As a long lived species, there may be a tradeoff in the energy that female killer whales devote to survival and reproduction [34]; females that devote more energy toward reproduction may experience higher mortality later in life [35,36]. Second, we evaluated support for the grandmother hypothesis – specifically, whether post-reproductive females had positive effects on the survival of grandoffspring, the reproductive lifespan of their daughters, or the time interval between their daughters' births. Finally, we evaluated whether there were sufficient data to estimate maternal tradeoffs between survival and reproduction.

## Methods

Longitudinal sightings data were collected from two neighboring populations of fish-eating killer whales over the last 30 years, 1978–2007 [23,37,38]: the Northern and Southern Resident killer whales which inhabit the inland and nearshore waters of Washington state (USA) and British Columbia (Canada). These populations are discrete; they do not interbreed, and neither immigration nor emigration have been observed [24]. During annual photographic surveys, nearly every individual in the population has been recorded. Each individual has unique pigmentation, scars, and fin shapes, allowing us to track the survival and reproductive performance of each female over time. Although detailed age and birth data do exist for recent years, information on birth defects, still births, or mortality risk to pregnant females is not available. Due to low adult mortality [22], the majority of females in our study are expected to live beyond the onset of menopause.

**Figure 2:**
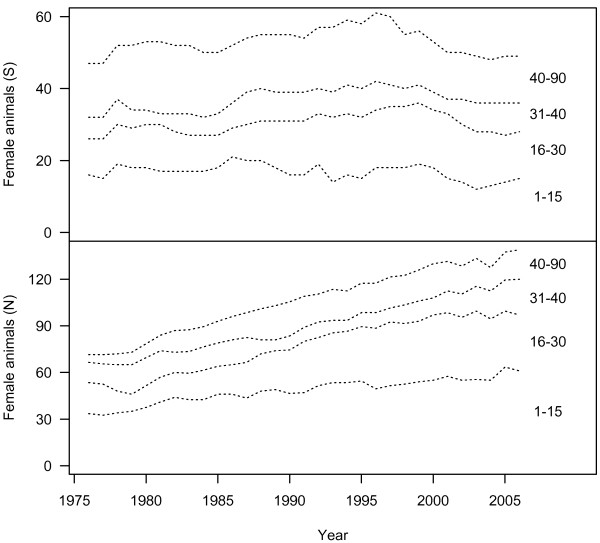
**Changes in the age-structure of female killer whales, from two populations since 1976**. Dashed lines are included for clarity to demarcate individuals aged 15, 30, and 40 years. After the 2007 survey, there are 49 females in the southern population (9 older than 45). Not all northern residents are seen each year, so there is some uncertainty in the exact age distribution – if all animals are alive, it is estimated that there are 139 females in the northern population (13 older than 45).

The age structure of females from the northern and southern populations was calculated to illustrate that over the last 30 years, each population has fluctuated in size, but more importantly, the age structure of both populations have changed (Fig. 2). In the smaller Southern Resident population, recruitment of young females has generally declined, while the proportion of older animals has remained relatively constant. The larger Northern Resident population appears to have increased in most years since the 1970s, and has shown an increase in the youngest component of the population (Fig. 2). The proportion of post-reproductive females has also fluctuated through time (Fig. 2), and it is unclear what role these animals may have in maintaining population structure.

To evaluate support for the attentive mother hypothesis, we examined whether covariate attributes of mothers (age, dead/alive, birth order) had any impact on the survival of calves (ages 0 to 1) or on juvenile survival (< age 5) for individuals born since 1978. Binomial generalized linear models (GLMs) with a logit link were used to model survival (*φ*) as a function of covariates, e.g. $\log\left(\frac{\phi }{1-\phi }\right)=B_{0}+B_{1}\cdot age_{mom}$. The Schwarz criterion or BIC [39] was calculated for each competing model, and differences between BIC values were used as an approximation to the Bayes factor [40]. If there are two competing models, the approximate Bayes factor supporting model 1 over model 2 is *BF*_12 _= exp [-(*BIC*_1 _- *BIC*_2_)/2]. When two models are considered, this formula allows the posterior probability of each model given the data to be computed, Pr [*M*_1_|*Y*] = *BF*_12_/(1+*BF*_12_), because the Bayes factor can also be viewed as the ratio of the posterior model probabilities.

To examine support for the grandmother hypothesis, we first looked at impacts of grandmothers on their daughters, and second evaluated potential impacts of grandmothers on grandoffspring survival. The reproductive lifespan of mature females was modeled using GLMs to evaluate support for including mothers' survival status as a covariate. Because the precise onset of reproductive termination cannot be determined, we defined the response variable (reproductive lifespan) as the number of years between the last and first birth. Only females whose mothers' survival status did not change over their reproductive histories were included in this calculation. An alternative effect grandmothers may have is that by providing additional care, daughters may be more productive (shortening the time between births). To examine support for this hypothesis, we constructed a model of interbirth intervals (IBI) as the response variable. The survival status of each females' mother (alive/dead) was included as a covariate to evaluate whether females with alive mothers had a shorter IBI compared to females with dead mothers. As the second component of evaluating the grandmother hypothesis, we expanded GLMs of survival to include the grandmothers' survival status as a covariate – in these models, we compared the age-specific survival rates of juveniles with living grandmothers to those with dead grandmothers. Two analyses were performed; in the first case we examined the impact of grandmothers on survival of calves (aged 0 to 1), in the second we examined this factor across juveniles (aged < 5).

One hypothesis for why survival of primiparous calves may be relatively low is that older mothers may be more experienced than younger mothers [41,42]. An alternative hypothesis is that there may be an evolutionary tradeoff between current survival and future reproduction – young females may prioritize their own survival above that of potential offspring, delaying reproduction [36]. To evaluate evidence supporting this hypothesis, we first had to quantify lifetime reproductive success (LRS) for each female. Several different approaches have been used to measure LRS. Lahdenpera et al. (2004) used the number of births as a response variable, however using the raw number of births has been criticized because it doesn't account for survival to maturity [14]. A second approach is to calculate the individual growth rate, *λ *[43] – this method has only been applied to short-lived species and cannot be applied to right-censored data, such as that in our study. A third approach involves estimating total recruits to the population, *R*_0 _[44]. This latter method may be more robust than calculating the individual *λ *because it is considered rate-insensitive [45].

**Figure 3:**
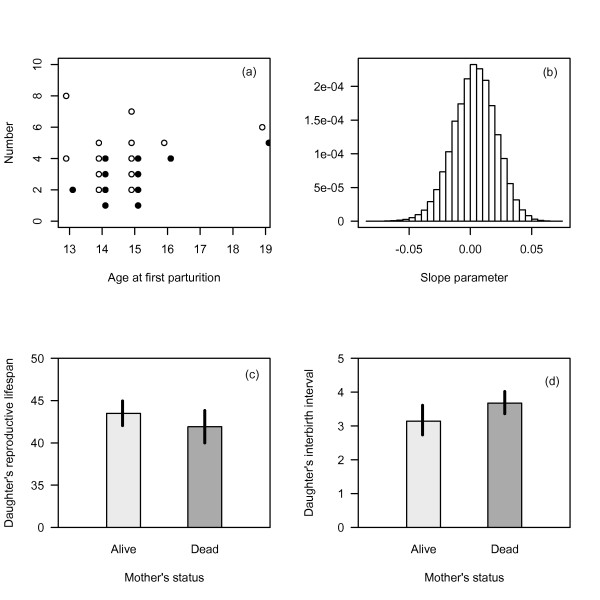
**Relationship between age at first parturition, offspring and 10-year old recruits (a), effect of senescent mothers on the reproductive lifespans and interbirth intervals of their daughters (b, c), and the simulated relationship between age at maturity and recruits produced for animals who haven't reached senescence (d)**. Open circles and light grey bars represent females whose mothers are alive. Females whose mothers' survival status changed over their reproductive lifespan were not included in panel (b). Solid vertical lines represent standard errors, complete sample sizes in Tables 1–2.

The total number of recruits could not be calculated for every animal in our study because many females either have partially observed reproductive histories or have not yet reached menopause. Instead, we developed a proxy for recruits, defining a recruit to be an offspring that lives to maturity (age 10) [22]. For each post-reproductive female whose entire reproductive history is known, we calculated the number of recruits, which was treated as a fixed constant (Fig. 3a). For females whose reproductive histories were incomplete, we performed Monte Carlo simulations to generate hypothetical distributions of future recruitment until the onset menopause. The reproductive performance of these individuals in the beginning of their reproductive lives may help inform potential tradeoffs between age at maturity and reproductive success; specifically, mothers that delay reproduction may produce more recruits. Three types of uncertainty were included in these simulations of future recruitment for these individuals (aged greater than 25): uncertainty in the future survival of the mother, uncertainty in the future reproductive performance of the mother, and uncertainty in the future survival of newborns to reproductive maturity. Estimates of natality from previous work were used as age-specific probabilities of producing calves [26]. Survival estimates of juveniles from the best model in our analysis (constant survival) were used with previously published estimates of adult survival for projecting individuals forward from birth [41]. Given the recruits per female at each iteration of the simulation, we used the known age at maturity for each female (age at first birth) to estimate the potential effect on the number of recruits. Recruits were modeled using Poisson GLMs, with age at maturity as a covariate: *R*_0_~*Poisson*(*u*), log(*u*) = *B*_0 _+ *B*_1 _· *age*_*mat*_.

## Results

### Support for the mutation accumulation hypothesis

Evolutionary theory predicts that if menopause is simply a byproduct of senescence, the difference between female lifespan and menopause would be approximately equal to the time necessary for a female to live long enough to raise her last offspring [12]. Killer whale fecundity declines gradually after age 23, and rapidly after age 40 (reproduction ceases by age 45) [22,26]. The difference between mean female life expectancy (55.1) and age at reproductive cessation (45) is approximately the same as the age at female maturity [41]. This result may be spurious if reproductive cessation results from the mutation accumulation or attentive mother hypotheses, but it also suggests that killer whale mothers may be similar to humans in that weaned juveniles may continue to be somewhat dependent on the presence of their mother, at least until they reach reproductive maturity.

### Support for the attentive mother hypothesis

When maternal age and birth order were separately considered as predictors of calf survival, a model that treated maternal age as a continuous predictor received less support than a model with no maternal effect (*Pr *[*M|Y*] = 0.19, Table 1). The effect of birth order on calf survival was also not supported (*Pr *[*M|Y*] = 0.17), possibly because maternal age and birth order are confounded. When maternal age was treated as a 2-level factor, including the effect of older mothers (> 35) did receive stronger support from the data (*Pr *[*M|Y*] = 0.64). Estimated survival rates for calves born to these older mothers were 10% higher than other calves, suggesting that the oldest mothers may be better mothers. When these same covariates were considered as predictors of juvenile survival (< age 5), there was little support for survival models including older mothers (*Pr *[*M|Y*] = 0.09) or birth order (*Pr *[*M|Y*] = 0.11). The lack of support for older mothers impacting juvenile survival suggests that the effect of older mothers may be only important during the first year of life.

**Table 1:** Models of calf and juvenile survival.

	*Calf survival*			*Juvenile survival*		
Model	BIC	*Pr *[*M|Y*]	*Pr *[*M|Y*] v. null	BIC	*Pr *[*M|Y*]	*Pr *[*M|Y*] v. null
null	177.7	0.29	-	419.4	0.14	-
age	-	-	-	416.9	0.51	0.78
age + age^2^	-	-	-	418.1	0.28	0.66
time	183.3	0.02	0.06	426.2	0.01	0.03
moms > 35 (F)	176.5	0.51	0.64	424.2	0.01	0.09
birth order	180.9	0.06	0.17	423.7	0.02	0.11
first born (F)	181.9	0.03	0.11	422.1	0.04	0.21
momAge	180.5	0.07	0.19	-	-	-
grandmaAlive	182.6	0.02	0.08	-	-	-

Models of calf survival (from age 0 to 1) were applied to 329 whales to evaluate maternal effects (binomial response) and for juvenile survival (ages 0 to 5) for 1411 whale-years (337 animals). In the latter comparison, juvenile survival was held constant (models that allowed survival to vary as a function of juvenile age were also considered, but the results did not change). All ages in the table refer to the age of the mother; birth order was treated as a continuous variable. Model selection tools like BIC are preferable over traditional significance tests, because they can be used to estimate model probabilities, *Pr *[*M|Y*]. For each model, we also calculated the probability for model *i *relative to the null model (constant survival, intercept only), *Pr *[*M*_*i*_*|Y*]/(*Pr *[*M*_*null*_*|Y*] + *Pr *[*M*_*i*_*|Y*]).

### Support for the grandmother hypothesis

**Figure 4:**
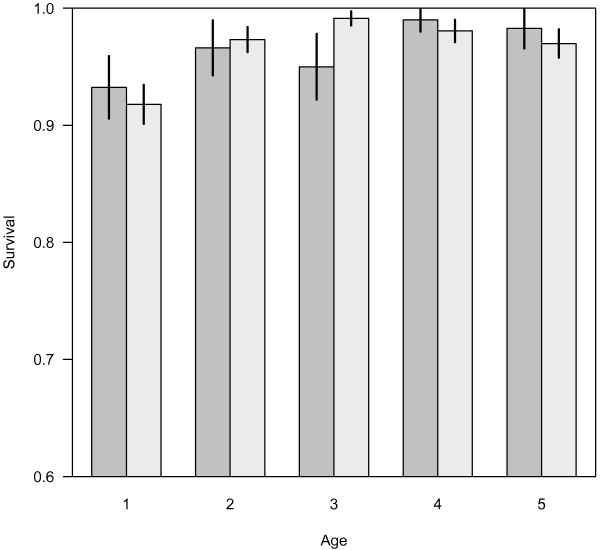
**Estimated age-specific juvenile survival rates, as a function of whether or not the grandmother is alive**. Dark bars represent animals whose grandmothers are known to be dead, light bars represent animals whose grandmothers are known to be alive (lines represent standard errors), complete sample sizes in Tables 1–2.

Females with living mothers had slightly longer reproductive lifespans than females without mothers, but the data did not provide strong support for including the mother's survival status in the model (Fig. 3b, *Pr *[*M|Y*] = 0.18, Table 2). Due to the small sample size, it is unclear whether grandmothers have no effect on their daughters' reproductive lifespans (Table 2). Additional care provided by grandmothers would be expected to decrease time intervals between births, however the effect of grandmothers on IBIs was not supported in our analysis (Fig. 3c, *Pr *[*M|Y*] = 0.35). A final effect we examined was whether post-reproductive grandmothers had positive effects on the survival of their grandoffspring. Across all ages, juvenile survival increased with age, and the presence of grandmothers did not appear to impact calf (*Pr *[*M|Y*] = 0.08) or juvenile survival (Fig. 4, *Pr *[*M|Y*] = 0.05). Despite the lack of support for including grandmothers across all ages, the biggest difference in estimated survival rates was for 3-year old animal (Fig. 4).

**Table 2:** GLM models illustrating how other hypotheses about killer whale reproduction were evaluated.

Comparison	Error	Model	n	n_A_	BIC	*Pr *[*M|Y*]
Repro. lifespan	Gaussian	Null	21		751.2	0.824
Repro. lifespan	Gaussian	mom's survival status	21	11	754.3	0.176
						
IBI	Poisson	Null	62		107.1	0.645
IBI	Poisson	mom's survival status	62	28	108.4	0.345
						
juvenile *φ*(1–5)	Binomial	Null	890		419.4	0.963
juvenile *φ*(1–5)	Binomial	grandma's survival status	890	674	426.6	0.027
juvenile *φ*(1–5)	Binomial	grandma's survival status + maternal age	890		432.9	0.001

This table includes comparisons for reproductive lifespan, females' interbirth intervals, and survival of grandoffspring (*φ*). In each case, the null model is the simplest possible (grandmothers have no effect). The sample size for each comparison (n) represents the number of whales, except in the case of juvenile survival, where it represents whale-years (an individual may be recorded for several years). The subset of sample size with grandmothers alive is also included (n_A_).

### Tradeoffs between survival and reproduction

Over all simulations, the distribution of the regression coefficient relating age at maturity to LRS was slightly positive (Fig. 3d), however the large variance prevents any conclusive inference about the relationship between age at maturity and reproduction (specifically, whether mothers that delay reproduction produce more viable calves later in life). Although three types of uncertainty were included in these projections, virtually no uncertainty existed in future survival of adult females (*φ*~0.99), and the largest source of variability was age-specific natality over ages 25–45 [26]. As these older animals reach menopause over the next 20 years, their reproductive performance will continue to provide more information about potential tradeoffs between age at maturity and lifetime reproductive success.

## Discussion

Hamilton (1966) first posited the idea of the "wall of death" – the idea that once females cease reproduction, mortality should increase rapidly as there is not selection pressure for continued survival [19]. We know that in human populations, there is little support for the "wall of death" hypothesis, even in hunter-gather populations where one cannot make the argument that survival past reproduction is due to artificially extended lifespan [46]. Until recently, it has been argued that menopause is specific to primates and that the "wall of death" still applies to non-primates. However, biologists have shown that menopause is a much more prevalent life-history trait in non-primates than was previously supposed [12,29]. As we show in this study, killer whales show even less support for the "wall of death" idea than human hunter-gatherer populations [46]. In these hunter-gatherer populations, approximately 30% of human females lived past reproduction; in contrast, more than 50% of killer whale females are expected to live beyond the onset of menopause (Fig. 1) [22,26].

Although there is some debate in the literature, we follow Cohen [29] and lump the attentive mother hypothesis and the helpful grandmother hypothesis into adaptive hypotheses for menopause. One explanation for the long post-reproductive lifespans that have been observed in killer whales is the presence (and potential helpfulness) of older matriarchal females within matrilineal units [33]. Using multiple modeling approaches, we found limited support for either of these adaptive explanations of menopause.

If the attentive mother hypothesis alone was responsible for the cessation of reproduction in killer whales, we would expect females to live just long enough to raise their last offspring to independence (2–3 years, to age 42–43). On average, female killer whales appear to live an average of 7–8 years longer than this (> 50 years, [22]) – roughly equivalent to the time needed for offspring to reach reproductive maturity. There is some uncertainty in the estimated life expectancy beyond menopause, however, due to the small sample size used and uncertainty in some ages of the oldest individuals [22]. For the attentive mother hypothesis to be responsible for menopause in killer whales, several biological conditions must be met: the probability of older females giving birth must decline with age, offspring born prior to the cessation of reproduction must experience some benefit, and the rates of birth defects, stillbirths and maternal mortality may increase with maternal age [47]. While the first two conditions appear to be met for this species ([26] and this study, respectively), data on stillbirths, birth defects, and maternal mortality do not exist. While it is unlikely that this data will be collected on killer whales or other free-ranging marine mammals, this question may be best explored in future research on terrestrial mammals with relatively long post-reproductive lifespans.

Although not directly related to the attentive mother hypothesis, we found that the oldest mothers may also be the best mothers – calves born to females approaching menopause had higher estimated survival rates than calves of younger mothers. These older females may be more successful in raising young because of maternal experience, or they may allocate more effort to reproduction relative to younger females. This same result did not hold when older females were used to model juvenile survival, suggesting that older mothers may be more experienced in caring for calves during their most dependent period (ages 0–1). After weaning, juveniles may depend on additional benefits from related individuals – fathers provide little to no parental care in this species, but older siblings or related adult females may contribute in raising young.

If the grandmother hypothesis was responsible for the cessation of reproduction in killer whales, either daughters or grandoffspring would be expected to benefit from the presence of post-reproductive females. From the perspective of the grandmother's fitness, the difference between these responses amounts to a tradeoff between the quantity and quality of grandoffspring produced. Post-reproductive females did not appear to have an effect on the number of calves produced by their daughters (either a shorter interbirth interval, longer reproductive lifespan or increased calving probability). Post-reproductive females also did not appear to benefit newborn grandoffspring, but following other studies, one of the age groups that may receive the largest benefit are newly weaned animals. In human studies, grandmothers have been shown to be one of the most common forms of kin selection [48]. An analysis of human menopause in 20^th ^century Gambia suggested that the benefit of grandmothers may occur during an extremely small temporal window, immediately after grandchildren are not completely dependent on their mothers [49]. The most compelling evidence supporting a positive benefit of killer whale grandmothers is the survival of grandoffspring between their 2^nd ^and 3^rd ^birthdays (Fig. 4). While including the survival status of grandmothers is not supported across all age classes, the posterior probability of the grandmother effect increases when only 2 year olds are included, but is still approximately equal to 50:50 support (*Pr *[*M|Y*] = 0.48 versus 0.05).

In a review of current evidence for post-reproductive lifespans in multiple taxa, Cohen's [29] non-adaptive hypothesis for the evolution of menopause suggests that there may be two forms of selection acting separately on reproductive and somatic lifespans. Cohen's hypothesis may explain the phenomenon of long post-reproductive lifespans in killer whales, but cannot be evaluated with existing data. To test this hypothesis using the longitudinal data in our study, we would need to examine whether total lifespans are uncorrelated with the length of reproductive lifespans [29]. Due to the extreme longevity of this species, females that were reaching reproductive maturity at the beginning of this study are currently reaching menopause, and are expected to live for another 15 years.

## Conclusion

In summary, like other studies of long-lived mammals [12,29], we found weak support for both of the adaptive hypotheses for menopause. Perhaps for anthropogenic reasons, the attentive mother and helpful grandmother hypotheses seem natural explanations for the presence of old females in social species – especially species that form multi-generation matriarchal groups. Aside from weak evidence in human populations, multiple studies have failed to find strong support for either of these adaptive hypotheses in other mammals. This does not mean that the hypothesis of "social transfer" [33] (the cultural transmission of knowledge and skills) or coalition formation [50] does not play a part in the evolution of menopause or the presence of old females in social groups. Rather, it suggests that if social transfers are playing a role, it is through more subtle effects than simply increasing the survival of daughters and granddaughters.

## Competing interests

The authors declare that they have no competing interests.

## Authors' contributions

EW performed the analyses and took the lead in writing the manuscript; KP and EH assisted in writing and revising the manuscript, as well as in developing the framework for the analysis; KB provided data and assisted with the initial editing and manuscript preparation; JF provided data and expertise in killer whale ecology, and detailed knowledge of problems in working with these datasets.
